# Prediction of Genetic Resistance for Scrapie in Ungenotyped Sheep Using a Linear Animal Model

**DOI:** 10.3390/genes12091432

**Published:** 2021-09-17

**Authors:** Mohammed Boareki, Flavio Schenkel, Delma Kennedy, Angela Cánovas

**Affiliations:** 1 Centre for Genetic Improvement of Livestock, Department of Animal Biosciences, University of Guelph, 50 Stone Road East, Guelph, ON N1G 2W1, Canada; schenkel@uoguelph.ca (F.S.); acanovas@uoguelph.ca (A.C.); 2 Ontario Ministry of Agriculture, Food and Rural Affairs, 6484 Wellington Road 7, Elora, ON N0B 1S0, Canada; delma.kennedy@ontario.ca

**Keywords:** sheep, scrapie resistance, BLUP, selection response, prediction accuracy

## Abstract

Selection based on scrapie genotypes could improve the genetic resistance for scrapie in sheep. However, in practice, few animals are genotyped. The objectives were to define numerical values of scrapie resistance genotypes and adjust for their non-additive genetic effect; evaluate prediction accuracy of ungenotyped animals using linear animal model; and predict and assess selection response based on estimated breeding values (EBV) of ungenotyped animals. The scrapie resistance (SR) was defined by ranking scrapie genotypes from low (0) to high (4) resistance based on genotype risk groups and was also adjusted for non-additive genetic effect of the haplotypes. Genotypes were simulated for 1,671,890 animals from pedigree. The simulated alleles were assigned to scrapie haplotypes in two scenarios of high (SR_h_) and low (SR_l_) resistance populations. A sample of 20,000 genotyped animals were used to predict ungenotyped using animal model. Prediction accuracies for ungenotyped animals for SR_h_ and SR_l_ were 0.60 and 0.54, and for allele content were from 0.41 to 0.71, respectively. Response to selection on SR_h_ and SR_l_ increased SR by 0.52 and 0.28, and on allele content from 0.13 to 0.50, respectively. In addition, the selected animals had large proportion of homozygous for the favorable haplotypes. Thus, pre-selection prior to genotyping could reduce genotyping costs for breeding programs. Using a linear animal model to predict SR makes better use of available information for the breeding programs.

## 1. Introduction

In the typical form of scrapie, the risk of infection is determined by variation in amino acid sequence encoded in the *PrP gene* [1,2,3,4]. There are five common haplotypes (ARR, AHQ, ARH, ARQ, and VRQ) associated with the scrapie risk of infection; in which the haplotype ARR is associated with lowest risk, and the haplotype VRQ is associated with highest risk of scrapie infection [2,5,6,7]. Other additional haplotypes have been observed in sheep, but due to their extreme rarity were not considered important for breeding programs [2,8]. A total of 15 common possible genotype combinations are associated with five risk groups (R1, R2, R3, R4, and R5), in which R1 genotypes are associated with low risk for scrapie infection (i.e., the most favorable genotypes) and R5 are associated with highest risk of infection [3,8,9]. Due to the association of genotypes with the risk of scrapie, the use of genotyping for breeding programs is appealing for scrapie eradication programs [5,8,10,11]. However, in practice, not all animals are being genotyped with breeding rams, which are more likely to be genotyped than ewes. Thus, genotypic information is limited, as only a small fraction of the total sheep population is genotyped. Gengler et al. [12] proposed the use of a practical method to predict the allele content of bi-allelic locus in ungenotyped animals by using the Best Linear Unbiased Predictor (BLUP), in which the number of observed alleles in the genotype (0, 1, or 2) are used as a response variable, assuming complete heritability.

Selection based on risk group of the genotypes could be practiced [3,5,8,9]. However, the genotypes corresponding to the risk groups do not act additively. For animal breeding purposes, the additive genetic effect is important since offspring inherit the alleles, rather than the genotype. There is no study that accounts for the non-additivity of risk groups corresponding to the genotypes. Therefore, adjusting for the non-additive effect is needed to account for the differences in contribution of the five different haplotypes alleles to scrapie resistance in sheep.

The objectives of this research were to: (1) define numeric values of scrapie resistance genotypes and adjust them for their non-additive genetic effect; (2) evaluate the accuracy of using BLUP for prediction of scrapie resistance and allele content of ungenotyped animals; and (3) predict selection response and assess the change of genetic merit of selected ungenotyped animals based on estimated breeding value. The hypothesis of this research was that it is possible use a linear animal model for genetic evaluation and selection of ungenotyped sheep for scrapie resistance based on a small proportion of genotyped animals.

## 2. Materials and Methods

### 2.1. Adjusting the Genetic Resistance to Scrapie for Non-Additive Genetic Effect

The numeric value of scrapie resistance genotypes was defined by ranking the scrapie genotypes from 0 (most susceptible) to 4 (most resistant), which were based on risk levels previously presented in literature [3,8,9] (see Table 1). The additive genetic effect of a haplotype was calculated as half of the homozygous resistance value relative to the most susceptible haplotype (VRQ), which was set to 0. The assumed additive genetic effect for scrapie resistance (SR) for each haplotype is shown in Table 1, along with the adjusted scrapie resistance (SR) genotypes for the non-additive genetic effects for all possible 15 genotypes, which were created by summing the additive genetic effects of the haplotypes. This step is important, as non-additive genetic effects were present. For example, the most susceptible haplotype VRQ is completely dominant over the haplotypes ARH and ARQ. Thus, genotypes VRQ/VRQ, ARH/VRQ, and ARQ/VRQ, have the same scrapie resistance level equal to 0 and are in the same risk group of R5, which is different from the risk group for the homozygous haplotypes ARH and ARQ. For animal breeding and genetic improvement purposes, additive genetic effects are more important than the non-additive genetic effects, as are transmitted to the next generation. Therefore, SR needs to be adjusted for non-additive genetic effects.

### 2.2. Simulated Data

A pedigree containing 1,671,890 sheep from the GenOvis database (www.genovis.ca; Guelph, ON, Canada accessed on 9 June 2017) was used to simulate genotypes at a single locus with five alleles resulting in 15 possible common genotypes. The genotype frequencies resulting from the simulation are shown in Table 2. The simulated alleles were assigned to scrapie haplotypes to create two population, high-SR, or low-SR, assuming high and low frequencies for the most resistant haplotype (ARR), respectively (Table 3). A total of 20,000 animals were randomly chosen (out of 1,671,890 individuals) to have genotype records in the study with all other animals assumed to be ungenotyped. Basic descriptive statistics of SR (0–4) in the two populations of high and low SR (SR_h_ and SR_l_, respectively) and the allele content (0, 1, or 2) for the haplotypes (Hc_1_, Hc_2_, Hc_3_, Hc_4_, and Hc_5_) are presented in Table 4 and Table 5, for whole population (*n* = 1,671,890) and the randomly chosen animals (*n* = 20,000) that had their genotypic information available (tested animals), respectively.

### 2.3. Prediction of Scrapie Resistance and Haplotype Gene Content Using the Animal Model

The scrapie resistance phenotypes (SR_h_ and SR_l_) and haplotype allele contents (Hc_1_, Hc_2_, Hc_3_, Hc_4_, and Hc_5_) where predicted for the ungenotyped animals using the observed records of the 20,000 genotyped animal using ASREML [13] and the following univariate linear animal model:

(1) $$y_{i}=µ+\;a_{i}+\;e_{i}$$

where, $y_{i}$: record of the *i*th animal for the trait being analyzed (SR_h_, SR_l_, Hc_1_, Hc_2_, Hc_3_, Hc_4_, or Hc_5_); µ: Overall mean trait $a_{i}$: additive genetic effect of the *i*th animal; and $e_{i}$: residual error.

The variance and covariance matrix for the univariate analysis was:

$$\left[\begin{array}{cc} A\sigma_{a}^{2} & 0\\ 0 & I\sigma_{e}^{2} \end{array}\right]$$

where, $\sigma_{a}^{2}$: is the additive genetic variance for the analyzed trait; $\sigma_{e}^{2}$: is the residual variance; A is the additive relationship matrix; and I is an identity matrix.

The heritability was assumed to be almost complete (h^2^ = 0.99), thus assuming a negligible residual. In order to estimate breeding values (EBV) for all animals (including the ungenotyped with no genotyped relatives) in the pedigree, unknown parents were assigned to genetic group based on sex and breed.

Accuracy of prediction for the ungenotyped individuals was evaluated using Pearson’s correlation between the EBV for SR or haplotype allele content and the true genetic value for all ungenotyped animals.

### 2.4. Selection Response

The responses to selection were predicted for ungenotyped animals by assuming that animals with breeding values ≥ mean would be selected, which makes the selection intensity equal to 0.798 [14], as follows:

$$R_{T}=0.798\times r_{T}\times \sigma_{aT}$$

where, $R_{T}$ is the predicted selection response for trait T (i.e., SR_h_, SR_l_ or haplotype allele contents (Hc_1_, Hc_2_, Hc_3_, Hc_4_, and Hc_5_); $r_{t,T}$ is thecorrelation between the predicted breeding value for the ungenotyped animals for trait T and the true genetic values for trait T; and $\sigma_{aT}$ is the additive genetic standard deviation for trait T, which is essentially equal to standard deviation of trait T, assuming a trait h^2^ = 0.99 (see Table 4).

The difference in genetic merit between the original population and selected animals that had EBV ≥ mean EBV was calculated for the ungenotyped animals as:

$$D_{T}={µ}_{Ts}-{µ}_{Tn}$$

where, $D_{T}\;$ is the difference in true genetic merit for trait T (i.e., SR_h_, SR_l_ or haplotype allele contents (Hc_1_, Hc_2_, Hc_3_, Hc_4_, and Hc_5_)) between selected (${µ}_{Ts}$) and unselected (${µ}_{Tn}$) animals.

In addition, selection based on EBV was performed at different selection truncation points (−1.282, −0.842, −0.525, −0.253, 0.000, +0.253, +0.525, +0.842, and +1.282) to assess: (1) the proportion of animals selected at different truncation point; (2) the proportion of recovered homozygous from ungenotyped animals among the selected animals (i.e., number of homozygous among the selected animals/number of homozygous among unselected animals) at different truncation point; (3) allele frequencies at different selection truncation points; and (4) homozygous genotype frequencies at different selection truncation points.

## 3. Results

### 3.1. Accuracies for Prediction of Scrapie Resistance and Haplotype Gene Content Using the Animal Model

The Pearson’s correlation coefficients between EBV and the trait true genetic value are shown in Table 6 for ungenotyped animals. The correlation between the EBV and the trait true genetic values (on diagonal) represents the accuracies of direct selection. Accuracies of prediction ranged from 0.41 to 0.71. Accuracy of predicting SR_h_ for ungenotyped individuals was high 0.60. In addition, the predicted SR_h_ was positively correlated (r = 0.58) with the most favorable haplotype in this analysis ARR (H_1_), and negatively correlated with the other haplotypes (H_2_, H_3_, H_4_, and H_5_ corresponding to AHQ, ARH, ARQ, and VRQ, respectively). The correlation between predicted allele content for ARR (Hc_1_) with its true content and SR_h_ was 0.71 and 0.47, respectively. This means that selection based on SR_h_ is expected to increase SR and ARR allele content, while decreasing the allele content for other haplotypes (i.e., AHQ, ARH, ARQ, and VRQ). On the other hand, prediction of SR_l_ was less accurate when predicting the true SR_l_ (r = 0.54) compared to SR_h_, and was negatively associated with the least favorable allele in this scenario, i.e., H_1_ (VRQ), while positively correlated with the other haplotypes (i.e., ARQ, ARH, AHQ, and ARR). This means that selection for SR_l_ will increase SR by replacing ARR by the other haplotypes in the population.

### 3.2. Selection Response

#### 3.2.1. Predicted Selection Response

Table 7 presents the predicted selection response per generation for scrapie resistance based on selecting animals with EBV ≥ mean EBV for SR_h_, SR_l_ and the haplotype allele contents (Hc_1_, Hc_2_, Hc_3_, Hc_4_, and Hc_5_) for ungenotyped animals (i.e., a selection intensity = 0.798). Selection of SR_h_ EBV was predicted to increase scrapie resistance by 0.46 and ARR (Hc_1_) allele content by 0.36, and decrease the other haplotype allele contents (i.e., for AHQ, ARH, ARQ, VRQ). Selection for Hc_1_ EBV was predicted to increase the SR_h_ by 0.36 and ARR (Hc_1_) allele content by 0.44, and decrease the other haplotypes allele contents. On the other hand, in the low resistance population (low-SR), direct selection for SR_l_ EBV was predicted to increase SR by 0.33 and increase the allele content of haplotypes ARQ, ARH, AHQ, and ARR, while decreasing the allele content of the most unfavorable haplotype VRQ. Compared to VRQ, selection for the other haplotypes was predicted to be slower, reflecting their lower accuracies of prediction (Table 6) and lower standard deviations (Table 5), which are dependent on the allele frequencies.

#### 3.2.2. Difference in Genetic Merit between Selected and Unselected Animals

Table 8 presents the difference in true genetic merit when animals were selected if their EBV was ≥ mean (selection intensity = 0.798). The true response was slightly different from the predicted response (Table 7). This shows that genetic improvement for SR is possible for ungenotyped animals using predictions from a linear animal model and the achieved improvement is similar to the predicted selection response. The difference in genetic merit when all animals were genotyped and selected based on true values (SR or allele contents) ≥ mean are presented on Table 9. In high-SR_h_ population, selection increased SR_h_ by 0.64 and increased the allele content of ARR (Hc_1_) and AHQ (Hc_2_) by 0.29 and 0.16, respectively. On the other hand, in a low-SR population, selection increased SR_l_ by 0.71 accompanied of a decrease in allele content of VRQ (Hc_1_) by 0.74, while increasing haplotype allele contents for ARQ (Hc_2_), ARH (Hc_3_), AHQ (Hc_4_), and ARR (Hc_5_) by 0.33, 0.13, 0.15, and 0.13, respectively. As expected, when all animals were genotyped, there is a higher gain than when only a fraction of the animals was genotyped for scrapie (Table 8). Table 10 presents the relative gain from having a fraction of genotyped animals compared to having all animals genotyped. The relative gain ranged between 13.2% and 95.0%, which is higher for higher allele frequency haplotypes than for low allele frequency haplotypes and higher for high-SR population compared low-SR population. Likewise, in the case of haplotype allele content, the relative gain was higher for high allele frequencies than for low allele frequencies. In practice, not all animals are being genotyped. Therefore, using an animal model can be practical and beneficial by adding extra information in an eradication program for scrapie in sheep. In addition, even considering the lowest relative gain of 13.2% (Table 10), it still would be beneficial, as the number of animals genotyped were a fraction of the whole population (i.e., 20,000 out of 1,671,890 animals).

#### 3.2.3. Effect of Selection at Different Selection Truncation Points

**Figure 1:**
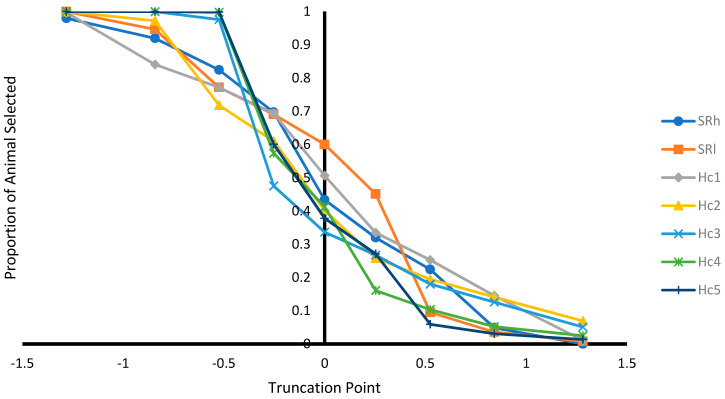
Proportion of animals selected from population at different selection truncation points in unit of standard deviations.

**Figure 2:**
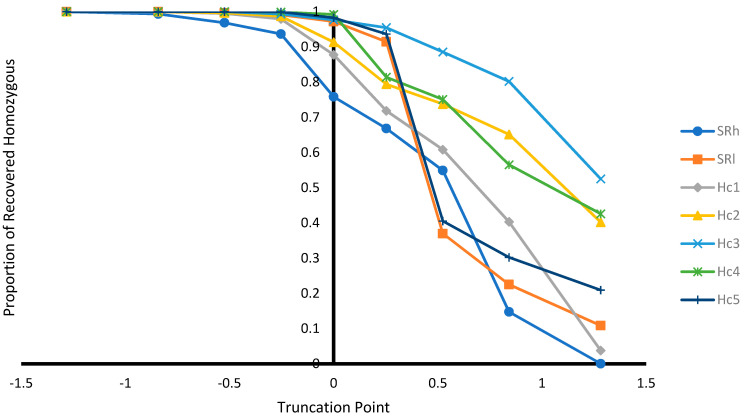
Proportion of recovered homozygous genotypes from the unselected population at different selection truncation points in unit of standard deviations.

**Figure 3:**
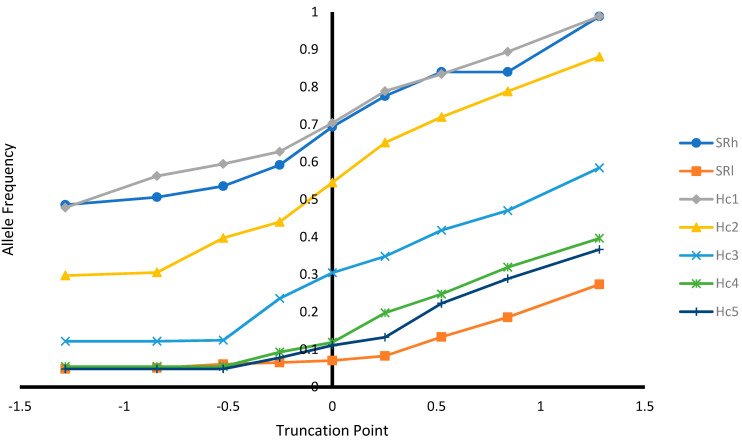
Allele frequency at different selection truncation points in unit of standard deviations.

**Figure 4:**
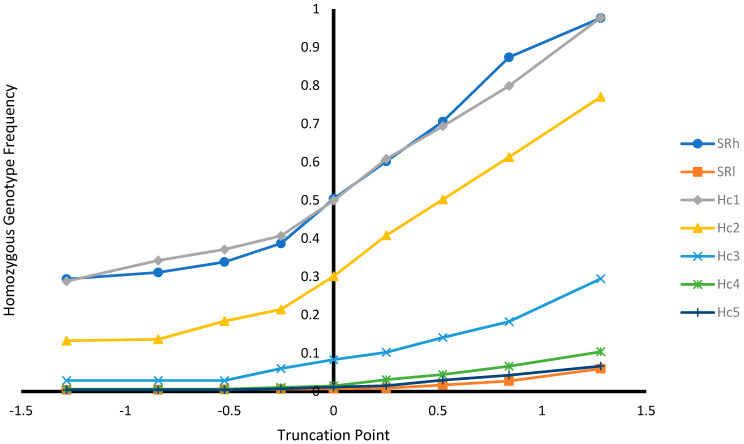
Homozygous genotype frequency at different selection truncation points in unit of standard deviations.

As expected, as the selection truncation point increased, the proportion of selected (Figure 1) and the recovered homozygous from the ungenotyped population (Figure 2) for the targeted haplotype allele decreased. On the contrary, as the selection truncation point increased, the allele frequency (Figure 3) and the homozygous frequency (Figure 4) for the targeted haplotype allele increased. Selecting animals based on their EBV being ≥ mean EBV resulted in selecting between 33.6% and 60.0% individuals (Figure 1) and recovering between 75.8 to 99.2% of homozygous genotypes (Figure 2). Assuming H_1_ as the favorable haplotype allele (i.e., ARR), selection based on Hc_1_ EBV ≥ mean EBV resulted in selecting 50.7% from the population and recovering a total of 87.7% of the target homozygous genotype (ARR/ARR). This means that by pre-selecting 50.7% of individuals based on EBV for allele content for genotyping, 87.7% of homozygous genotypes could be captured from the original population. Thus, this would reduce the number of animals required for genotyping. In the case of low ARR allele frequency (i.e., H_5_), when pre-selecting based on EBV for allele content ≥ mean EBV, the number of individuals required for genotype validation was reduced to 37.8% (Figure 1), while recovering 98.3% (Figure 2) of homozygous genotype (ARR/ARR) from the original population. Thus, the genotyping cost could be reduced even more.

## 4. Discussion

The accuracy for prediction of haplotype allele contents ranged between 0.41 and 0.71 (Table 6). Gengler et al. [12], who first proposed the allele content model, used it for the *myostatin gene* in Belgium blue cattle, with prediction accuracies between 0.47 and 0.50. In another study in Canadian Holstein cattle, the prediction accuracy for allele content was as high as 0.93 [15]. Legarra and Vitezica [16] reported accuracies between 0.52 and 0.56 using the allele content model. The allele content model can be applicable for prediction in bi-allelic major *genes*, such as for maedi-visna [17] and for litter size [18]. Applying allele content model to scrapie as first proposed by Gengler et al. [12] is possible when only considering the number ARR haplotypes and disregarding the importance of the other haplotypes. However, the *PrP gene* underlying scrapie phenotypes is multi-allelic with different contributions from the different haplotypes to the level of scrapie resistance in sheep [3,8,9]. In this study, the different contributions of haplotypes in the SR genotypes were considered by defining numeric values for SR and adjusting them to non-additive genetic effects prior to their use in the linear animal model. The accuracy for prediction of SR in high and low SR populations were 0.60 and 0.54, respectively. There were no previous studies that considered the same approach to construct the SR values to compare to.

Selection response on SR and allele content was predicted for ungenotyped sheep (Table 7). The selection response depends on selection intensity, accuracy, and the additive genetic standard deviation [14]. The differences between the trait prediction accuracies (Table 6) and the additive genetic standard deviations (Table 4) explain the differences in predicted responses. Selecting ungenotyped animals with EBV ≥ mean resulted in increase in genetic merit for SR and allele content (Table 8) and increased favorable haplotype allele frequency (Figure 3). Breeding programs for genetic improvement for SR involve selection based on scrapie genotypes. Such breeding programs were successful in increasing ARR frequency and SR in Czech Republic [8], Netherland [19,20], Belguim [21], and Hungary [5]. However, in all previous studies, the genetic change was limited to the animals being genotyped. In this research, the genetic improvement for SR was possible for the ungenotyped animals when using predictions from a linear animal model.

Selecting ungenotyped animals with EBV ≥ mean resulted in reduction of number of animals compare to unselected population (Figure 1), while capturing large proportion of homozygous genotypes from the unselected population (Figure 2). The increase of selection truncation point resulted in increased frequency for homozygous genotypes (Figure 4), while decreasing the proportion of animals selected from the population (Figure 1). In the breeding program in Netherlands, genotyping for selection of ARR/ARR rams was compulsory between the year 2004 and 2007 [20]. Genotyping to identify homozygous (ARR/ARR) rams is important for breeding purposes, as they are 100% guaranteed to transmit the ARR allele to their progeny. The use of linear animal model provides another tool to reduce the number of genotyped animals by pre-selecting based on EBV prior to genotyping. The pre-selected animals with EBV ≥ mean include a large proportion of ARR/ARR genotypes from the unselected population (Figure 2). Without pre-selection, 100% of the animals in the population must be genotyped in order to identify all the ARR/ARR animals in the population. When animals with EBV ≥ mean were pre-selected, smaller proportion (34–60%) would need to be genotyped in order to identify a large proportion (76–99%) of ARR/ARR animals (Figure 1 and Figure 2). Thus, genotyping cost for identifying most of the ARR/ARR animals in the population could be reduced. As pre-selection truncation point increases, the proportion of animal selected decreases (Figure 1), but the frequency of ARR/ARR animals among the selected animals increases (Figure 4). Thus, pre-selecting at higher truncation point would identify large proportion of ARR/ARR among the selected animals, thus saving genotyping cost to confirm the homozygous ARR/ARR status of the animals.

Breeding programs can contribute to the reduction of prevalence of the typical form of scrapie in sheep. Arnold and Rajamyagam [22] estimated an annual reduction of 28% in scrapie prevalence cases between 2005 and 2019 in Great Britain. Hagenaars et al. [20] reported a trend for reduction of scrapie prevalence in active scrapie surveillance in Netherland between the years 2002 and 2008. They reported 0% typical scrapie cases in genotypes with risk levels R1 and R2. The current study showed that selecting ungenotyped sheep based on EBV could increase SR (Table 7 and Table 8) and ARR allele frequency and its homozygous genotype frequency (Figure 3 and Figure 4), what could reduce typical scrapie prevalence in sheep. However, the atypical form of scrapie (Nor98) could occur in sheep that are resistant to typical scrapie [23]. However, Nor98 scrapie type is believed to be a spontaneous disease and it is unlike to be naturally contagious among sheep and, thus, its prevalence is low [24,25]. Therefore, a well-established breeding program for the typical scrapie can contribute to the reduction of scrapie prevalence.

This research proposed the use of a linear animal model as a practical method for genetic evaluation and selection for SR of ungenotyped sheep. Different scrapie eradication strategies used in breeding programs were described in previous studies. For instance, Arnold et al. [26] proposed genotyping purebred rams in the nucleus flocks used for cross-breeding and selecting the homozygous ARR/ARR and the carriers (ARR/ARQ, ARR/ARH, and ARR/AHQ) rams at the pure breeding level. Molina et al. [3] compared different strategies in Spanish Merinos. They concluded that the optimum strategy was to genotype rams and eliminate ARQ/ARQ and VRQ carriers. According to Gáspárdy et al. [5], in the Hungarian national breeding program, rams are genotyped and only rams at risk groups (R1, R2, and R3) are allowed to breed. In all previous studies, the genetic selection for SR was limited to the animals genotyped. This study has shown that a linear animal model can be used to provide additional information for ungenotyped animals, which will be particularly useful wherever genotyping for scrapie is not intensively practiced. Therefore, the use of an animal model could make better use of the available information to enhance breeding programs for the genetic improvement for SR in sheep.

## 5. Conclusions

Moderate to highly accurate estimated breeding values for scrapie resistance and haplotype allele content for ungenotyped animals can be obtained from a linear animal model using genotype data from only a fraction of the total sheep population. Thus, selecting ungenotyped animals based on EBV could result in effective genetic gains for scrapie resistance and allele content. Individuals with EBV ≥ mean were shown to carry a large proportion of homozygous genotypes. Thus, pre-selection prior to genotyping could reduce the number of animals needed to be genotyped to identify individuals with the favorable homozygous genotypes in the population and, consequently, reduce the genotyping cost. Therefore, a linear animal model could make better use of the available information for genetic improvement of scrapie resistance in sheep.

## Figures and Tables

**Table 1:** Risk groups, unadjusted and adjusted scrapie resistance genotypes, and additive genetic values for scrapie resistance for each haplotype.

Genotypes	Risk Goup ^1^	Unadjusted SR ^2^	Adjusted SR ^3^
ARR/ARR	R1	4	4
ARR/AHQ	R2	3	3.5
AHQ/AHQ	R2	3	3
ARQ/AHQ	R3	2	2
AHQ/ARH	R3	2	2
ARR/ARH	R3	2	2.5
ARR/ARQ	R3	2	2.5
AHQ/VRQ	R4	1	1.5
ARR/VRQ	R4	1	2
ARQ/ARQ	R4	1	1
ARQ/ARH	R4	1	1
ARH/ARH	R4	1	1
ARH/VRQ	R5	0	0.5
ARQ/VRQ	R5	0	0.5
VRQ/VRQ	R5	0	0
**Haplotype**	**Additive genetic value ^4^**		
ARR	2		
AHQ	1.5		
ARH	0.5		
ARQ	0.5		
VRQ	0		

^1^ Risk group: genotype risk group to scrapie from low (R1) to high (R5) risk; ^2^ Unadjusted SR: unadjusted numeric values for scrapie resistance genotypes from low (0) to high (4) scrapie resistance; ^3^ Udjusted SR: adjusted scrapie resistance genotypic value for a non-additive genetic effects, by adding the additive genetic effects of the haplotypes; ^4^ additive genetic value for haplotypes.

**Table 2:** Genotype count and frequencies in the simulation.

Genotype ^1^	Count	Frequency
H_1_/H_1_	482,112	0.2884
H_1_/H_2_	334,271	0.1999
H_1_/H_3_	144,469	0.0864
H_1_/H_4_	79,958	0.0478
H_1_/H_5_	75,690	0.0453
H_2_/H_2_	221,928	0.1327
H_2_/H_3_	126,363	0.0758
H_2_/H_4_	41,457	0.0248
H_2_/H_5_	48,086	0.0288
H_3_/H_3_	48,011	0.0287
H_3_/H_4_	29,460	0.0176
H_3_/H_5_	11,376	0.0068
H_4_/H_4_	10,224	0.0061
H_4_/H_5_	11,188	0.0067
H_5_/H_5_	7297	0.0044

^1^ Genotypes at single locus resulting from simulation of multi-allelic locus with five different haplotypes (H_1_–H_5_).

**Table 3:** Genotypes assigned to haplotypes for the scrapie resistance analyses ^1^.

Haplotype ^2^	SR_h_	SR_l_
H_1_	ARR	VRQ
H_2_	AHQ	ARQ
H_3_	ARH	ARH
H_4_	ARQ	AHQ
H_5_	VRQ	ARR

^1^ Two populations of different levels of scrapie resistance. Where, SR_h_ has high frequency for ARR haplotype, while SR_l_ has very low frequency for ARR haplotype. ^2^ Haplotypes simulated in the study, for which frequencies from Table 2 and unadjusted SR values from Table 1 were assigned.

**Table 4:** Descriptive statistics for all the individuals in the population.

Trait ^1^	Range	Mean ± SD	CV%
SR_h_	0–4	2.98 ± 0.96	32.10
SR_l_	0–4	0.78 ± 0.77	99.45
Hc_1_	0–2	0.96 ± 0.79	82.26
Hc_2_	0–2	0.59 ± 0.71	119.71
Hc_3_	0–2	0.24 ± 0.49	201.66
Hc_4_	0–2	0.11 ± 0.33	303.10
Hc_5_	0–2	0.10 ± 0.31	321.41

^1^ SR_h_ and SR_l_ are the scrapie resistance traits (SR) in a high SR population and a low SR population (see Table 3); and Hc_1_–Hc_5_ are the haplotype allele contents, i.e., the number of a given scrapie haplotypes observed in the genotype (0, 1, or 2).

**Table 5:** Descriptive statistics for the 20,000 individuals sampled with genotypic records used for analysis.

Trait ^1^	Range	Mean ± SD	CV%
SR_h_	0–4	2.98 ± 0.95	32.01
SR_l_	0–4	0.78 ± 0.77	99.04
Hc_1_	0–2	0.95 ± 0.79	82.54
Hc_2_	0–2	0.60 ± 0.71	119.04
Hc_3_	0–2	0.24 ± 0.49	203.65
Hc_4_	0–2	0.11 ± 0.33	299.49
H_5_	0–2	0.10 ± 0.31	318.71

^1^ SR_h_ and SR_l_ are the scrapie resistance traits (SR) in a high SR population and a low SR population (see Table 3); and Hc_1_–Hc_5_ are the haplotype allele contents, i.e., the number of a given scrapie haplotypes observed in the genotype (0, 1, or 2).

**Table 6:** Correlation coefficients ^1^ (accuracies) between predicted breeding values for SR or for haplotype allele content and their corresponding true values in ungenotyped individuals.

	True						
Predicted	SR_h_	SR_l_	Hc_1_	Hc_2_	Hc_3_	Hc_4_	Hc_5_
**SR_h_**	**0.602**	−	0.576	−0.167	−0.454	−0.240	−0.102
**SR_l_**	−	**0.537**	−0.552	0.290	0.136	0.264	0.237
**Hc_1_**	0.478	−0.416	**0.707**	−0.537	−0.278	−0.079	−0.036
**Hc_2_**	−0.139	0.217	−0.536	**0.702**	−0.070	−0.128	−0.003
**Hc_3_**	−0.430	0.121	−0.317	−0.081	**0.646**	0.087	−0.129
**Hc_4_**	−0.300	0.307	−0.123	−0.187	0.103	**0.484**	0.060
**Hc_5_**	−0.140	0.300	−0.052	−0.012	−0.198	0.059	**0.412**

^1^ The correlation between the prediction on the same trait (SR_h_, SR_l_, Hc_1_, Hc_2_, Hc_3_, Hc_4_, or Hc_5_; on diagonal and bold) and between the prediction on the different traits (off diagonal). SR_h_ and SR_l_ are the scrapie resistance traits (SR) in a high SR population and a low SR population (see Table 3), and Hc_1_–Hc_5_ are the haplotype allele contents, i.e., the number of a given scrapie haplotypes observed in the genotype (0, 1, or 2).

**Table 7:** Predicted selection response ^1^ from the same trait (on diagonal and bold) and from different traits ^2^ (off diagonal) in ungenotyped individuals.

	Response on						
Selection on	SR_h_	SR_l_	Hc_1_	Hc_2_	Hc_3_	Hc_4_	Hc_5_
**SR_h_**	**0.459**	−	0.362	−0.095	−0.177	−0.063	−0.025
**SR_l_**	−	**0.331**	−0.347	0.165	0.053	0.070	0.059
**Hc_1_**	0.364	−0.256	**0.444**	−0.305	−0.108	−0.021	−0.009
**Hc_2_**	−0.106	0.133	−0.337	**0.399**	−0.027	−0.034	−0.001
**Hc_3_**	−0.327	0.075	−0.199	−0.046	**0.252**	0.023	−0.032
**Hc_4_**	−0.229	0.189	−0.077	−0.106	0.040	**0.128**	0.015
**Hc_5_**	−0.106	0.185	−0.032	−0.007	−0.077	0.016	**0.102**

^1^ Response to selection based on predicted breeding values and assuming intensity = 0.798. ^2^ Traits are SR_h_, SR_l_, Hc_1_, Hc_2_, Hc_3_, Hc_4_, and Hc_5_, where SRh and SR_l_ are the scrapie resistance traits (SR) in a high SR population and a low SR population (see Table 3), and Hc_1_–Hc_5_ are the haplotype allele contents, i.e., the number of a given scrapie haplotypes observed in the genotype (0, 1, or 2).

**Table 8:** True selection response ^1^ from the same trait (on diagonal and bold) and from different traits ^2^ (off diagonal) in ungenotyped individuals.

	Response On						
Selection on	SR_h_	SR_l_	Hc_1_	Hc_2_	Hc_3_	Hc_4_	Hc_5_
**SR_h_**	**0.515**	−	0.432	−0.159	−0.145	−0.075	−0.052
**SR_l_**	−	**0.284**	−0.332	0.185	0.048	0.052	0.045
**Hc_1_**	0.397	−0.271	**0.452**	−0.284	−0.126	−0.036	−0.001
**Hc_2_**	−0.180	0.193	−0.450	**0.496**	−0.013	−0.037	0.003
**Hc_3_**	−0.509	0.143	−0.356	−0.001	**0.366**	0.044	−0.052
**Hc_4_**	−0.261	0.246	−0.077	−0.119	0.014	**0.129**	0.053
**Hc_5_**	−0.094	0.232	−0.024	0.005	−0.139	0.031	**0.126**

^1^ Response to selection calculated based on estimated breeding values (EBV). ^2^ Traits are SR_h_, SR_l_, Hc_1_, Hc_2_, Hc_3_, Hc_4_, and Hc_5_, where SRh and SR_l_ are the scrapie resistance traits (SR) in a high SR population and a low SR population (see Table 3), and Hc_1_–Hc_5_ are the haplotype allele contents, i.e., the number of a given scrapie haplotypes observed in the genotype (0, 1, or 2).

**Table 9:** True selection response ^1^ from the same trait (on diagonal and bold) and from different traits ^2^ (off diagonal) when all animals in the populations are genotyped (*n* = 1,671,890).

	Response On						
Selection on	SR_h_	SR_l_	Hc_1_	Hc_2_	Hc_3_	Hc_4_	Hc_5_
**SR_h_**	**0.645**	−	0.294	0.155	−0.244	−0.109	−0.096
**SR_l_**	−	**0.711**	−0.737	0.333	0.126	0.148	0.130
**Hc_1_**	0.433	−0.318	**0.476**	−0.295	−0.114	−0.038	−0.028
**Hc_2_**	−0.075	0.155	−0.523	**0.693**	−0.08	−0.055	−0.034
**Hc_3_**	−1.043	0.153	−0.555	−0.243	**0.890**	−0.027	−0.065
**Hc_4_**	−1.076	1.149	−0.492	−0.354	−0.073	**0.950**	−0.031
**Hc_5_**	−1.453	1.622	−0.464	−0.282	−0.170	−0.036	**0.951**

^1^ Response to selection calculated based on estimated breeding values (EBV). ^2^ Traits are SR_h_, SR_l_, Hc_1_, Hc_2_, Hc_3_, Hc_4_, and Hc_5_, where SR_h_ and SR_l_ are the scrapie resistance traits (SR) in a high SR population and a low SR population (see Table 3), and Hc_1_–Hc_5_ are the haplotype allele contents, i.e., the number of a given scrapie haplotypes observed in the genotype (0, 1, or 2).

**Table 10:** Relative gain in the true selection response when a fraction of the population was genotyped for scrapie (*n* = 20,000) relative to the response when the whole population (*n* = 1,671,890) was genotyped.

Trait ^1^	Relative Gain%
SR_h_	80.00
SR_l_	39.94
Hc_1_	94.96
Hc_2_	71.57
Hc_3_	41.12
Hc_4_	13.58
Hc_5_	13.25

^1^ Traits are SR_h_, SR_l_, Hc_1_, Hc_2_, Hc_3_, Hc_4_, and Hc_5_, where SR_h_ and SR_l_ are the scrapie resistance traits (SR) in a high SR population and a low SR population (see Table 3), and Hc_1_–Hc_5_ are the haplotype allele contents, i.e., the number of a given scrapie haplotypes observed in the genotype (0, 1, or 2).

## Data Availability

Not applicable.
